# Establishment of a long-term stable β-cell line and its application to analyze the effect of *Gcg* expression on insulin secretion

**DOI:** 10.1038/s41598-020-79992-7

**Published:** 2021-01-12

**Authors:** Satsuki Miyazaki, Fumi Tashiro, Takashi Tsuchiya, Kazuki Sasaki, Jun-ichi Miyazaki

**Affiliations:** 1 Division of Stem Cell Regulation Research, Center for Medical Research and Education, Osaka University Graduate School of Medicine, Suita, Osaka Japan; 2 National Cerebral and Cardiovascular Center, Suita, Osaka Japan; 3 The Institute of Scientific and Industrial Research, Osaka University, Ibaraki, Osaka 560-0047 Japan; 4 Present Address: Sasaki Institute, 2-2, Kandasurugadai, Chiyoda-ku, Tokyo, 101-0062 Japan

**Keywords:** Biochemistry, Biological techniques, Endocrinology

## Abstract

A pancreatic β-cell line MIN6 was previously established in our lab from an insulinoma developed in an IT6 transgenic mouse expressing the SV40 T antigen in β-cells. This cell line has been widely used for in vitro analysis of β-cell function, but tends to lose the mature β-cell features, including glucose-stimulated insulin secretion (GSIS), in long-term culture. The aim of this study was to develop a stable β-cell line that retains the characteristics of mature β-cells. Considering that mice derived from a cross between C3H and C57BL/6 strains are known to exhibit higher insulin secretory capacity than C57BL/6 mice, an IT6 male mouse of this hybrid background was used to isolate insulinomas, which were independently cultured. After 7 months of continuous culturing, we obtained the MIN6-CB4 β-cell line, which stably maintains its GSIS. It has been noted that β-cell lines express the glucagon (*Gcg*) gene at certain levels. MIN6-CB4 cells were utilized to assess the effects of differential *Gcg* expression on β-cell function. Our data show the functional importance of *Gcg* expression and resulting basal activation of the GLP-1 receptor in β-cells. MIN6-CB4 cells can serve as an invaluable tool for studying the regulatory mechanisms of insulin secretion, such as the GLP-1/cAMP signaling, in β-cells.

## Introduction

Type 2 diabetes is characterized by insufficient secretion of insulin from pancreatic β-cells. Understanding the physiological processes that regulate insulin production and secretion is essential for the development of type 2 diabetes-related drugs and treatment. Because of the limited availability of primary β-cells, immortalized rodent β-cell lines such as RIN^1^, HIT^2^, βTC^3^, MIN6^4^, NIT-1^5^, INS-1^6^, and βHC cells^7^ have been widely used to analyze these processes. However, these cell lines present vast differences in the amount of insulin secreted and their insulin-secretory response to glucose and other secretagogues^8,9^.

Among these β-cell lines, MIN6, βHC-9, and INS-1 retain normal glucose-stimulated insulin secretion (GSIS), while the others do not respond to glucose or respond at much lower glucose concentrations (< 5 mM) than normal islets. Studies have been conducted to clarify the cause of such inappropriate responsiveness to subphysiological concentrations of glucose. The results revealed that it is mainly due to the ectopic expression of a low-Km glucose-phosphorylating enzyme hexokinase I encoded by *Hk1* instead of the *Gck*-encoded high-Km isozyme glucokinase, which is the major isozyme in islet β-cells^10^. Moreover, overexpression of *Hk1* in *Gck*-expressing MIN6 cells resulted in insulin secretion at lower glucose concentrations^11^. Other groups reported that the glucose phosphorylating step, but not glucose transport step, regulates GSIS by modulating the glycolytic rate in β-cells^12,13^.

Although MIN6, βHC-9, and INS-1 cells retain GSIS, in long-term culture they show a tendency to lose the differentiated phenotype of β-cells, including insulin response to glucose^8,14^. It was probably due to the poorly glucose-responsive cells dominating in the culture or the change in expression of GSIS-responsible genes over time. To avoid this problem, stable subclones such as MIN6-m9^15^, MIN6B1^16^, MIN6-cl4^14^, and INS-1E^17^ were isolated and used for β-cell research. However, these subclones were not necessarily stable in long-term culture or they presented some gene expression abnormalities^15^. The aim of this study was to establish a β-cell line that is stable in long-term culture and shows gene expression similar to normal β-cells. Original MIN6 cells were established from an insulinoma developed in an IT6 transgenic C57BL/6 mouse line which expresses the SV40 T antigen under control of the human insulin promoter^4^. We have established a number of IT6-derived β-cell lines by crossing IT6 mice to various gene-knockout or transgenic mice^18–22^. These β-cell lines mostly retained GSIS, but their long-term stability has not been studied. Considering that C3H x C57BL/6 F1 (C3B6F1) mice are known to show higher insulin secretory capacity than C57BL/6 mice^23^, an IT6 male mouse of the C3B6F1 background was used to isolate insulinomas. From 50 insulinoma cell lines, 5 cell lines were chosen based on the stability of GSIS.

Glucagon-like peptide-1 (GLP-1) is a 30 or 31 amino acid peptide produced in the intestinal enteroendocrine L cells by differential processing of preproglucagon encoded by the *Gcg* (glucagon) gene^24^. GLP-1 potentiates GSIS through binding to the GLP-1 receptor highly expressed on the β-cell membrane^25^. The GLP-1 receptor is a member of the G protein–coupled receptor family and GLP-1 binding to this receptor activates adenylate cyclase and increases cyclic AMP (cAMP) levels under elevated glucose. Exendin-4, a 39 amino acid peptide isolated from the salivary glands of the Gila monster (*Heloderma suspectum*), acts as a GLP-1 receptor agonist^26^.

MIN6 cells express not only the insulin genes (*Ins1* and *Ins2*), but also the *Gcg* gene^27,28^. MIN6 cells were shown to secrete GLP-1 in response to glucose. GLP-1 secretion was further accelerated by the GLP-1 receptor agonist exendin-4, suggesting that MIN6 cells possess an autocrine mechanism for GLP-1 signal amplification^29^. Another mouse insulinoma cell line, β-TC-6, and rat insulinoma cell line, INS-1, were also reported to express the *Gcg* gene^30,31^. Newly established 5 cell lines expressed the *Gcg* gene at low, but varying levels.

In the present study, one of the newly established β-cell lines was utilized to examine the effects of differential expression of the *Gcg* gene on β-cell function. Based on the results, we discuss the possible significance of *Gcg* expression and basal activation of the GLP-1 receptor in β-cells of pancreatic islets.

## Results

### Establishment of stable pancreatic β-cell lines of the C3B6F1 background

**Figure 1:**
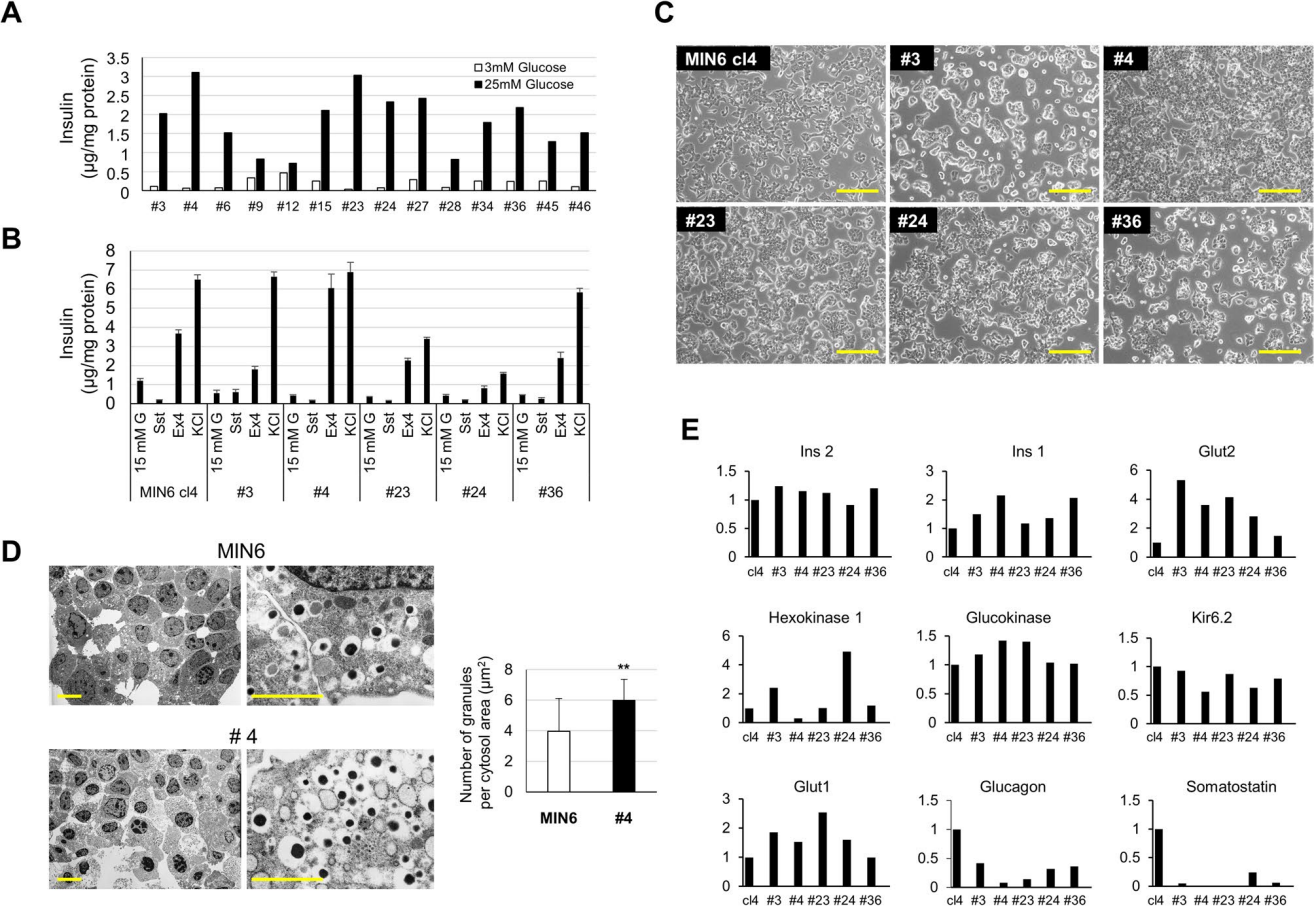
Stable pancreatic β-cell lines were selected from continuously cultured insulinoma cells derived from an IT6 transgenic male mouse on the C3B6F1 genetic background. (**A**) Insulin secretion of MIN6-CB cell lines stimulated with 3 or 25 mM glucose. Values represent means (n = 2). Five CB cell lines (#3, #4, #23, #24, and #36) were selected which showed proper GSIS after long-term culture. (**B**) Insulin secretion of MIN6-cl4 and CB cell lines (#3, #4, #23, #24, and #36) stimulated with 15 mM glucose alone or with somatostatin (Sst; 10 nM), exendin-4 (Ex4; 20 nM), or KCl (30 mM). Values represent means ± S.D. (n = 3). (**C**) Phase-contrast micrographs of MIN6-cl4 and CB cell lines (#3, #4, #23, #24, and #36). Some cell lines (cl4, CB24, and CB36) show neurite-like structures. Scale bars, 200 μm. (**D**) Transmission electron microscopy of original MIN6 and MIN6-CB4 cells. Scale bars, 5 μm (left panels), 1 μm (right panels). The right graph shows insulin granule density in the cytoplasm of MIN6 and CB4 cells. CB4 cells include more insulin granules than MIN6 cells. Values are means ± S.D. (n = 20). **P < 0.01 by Student’s t-test. (**E**) Quantitative RT-PCR analysis of β-cell-related genes in MIN6-cl4 and CB cell lines (#3, #4, #23, #24, and #36). Values represent means of technical duplicate.

We crossed the IT6 mouse of the C57BL/6 genetic background to the C3H mouse strain. C3H and C3B6F1 mice are known to show better capacity for glucose tolerance than C57BL/6 mice^23^. We isolated 50 insulinomas of the C3B6F1 genetic background and independently cultured them. The resulting cell lines were designated as MIN6-CB1 to -CB50. During the early stage of culture, 12 clones showed poor cell growth and/or fibroblast contamination and were removed. The other 38 clones were subjected to GSIS experiments at passage 8 (Fig. S1A). We selected 14 cell lines from them on the basis of homogeneous colony morphology and insulin secretory response to glucose. After continuous culture for 7 months, we chose five CB cell lines (#3, #4, #23, #24, and #36) which maintained proper insulin secretion in response to glucose (Fig. 1A). We examined insulin secretion by these CB cell lines after stimulation with 15 mM glucose alone and together with somatostatin, exendin-4, and KCl (Fig. 1B). As a control, we included MIN6-cl4, which is a subclone derived from parental MIN6 cells and retains stable phenotype^14^. CB23 and CB24 cell lines showed lower insulin secretion by KCl stimulation than the other cell lines. Somatostatin treatment reduced the insulin secretion in all these cell lines except for CB3 cells. Interestingly, insulin secretion in response to exendin-4 was variable, but CB4 cells showed the best response among all the cell lines tested.

Bright field images of the living MIN6-cl4 and CB cells are shown in Fig. 1C. Some of the cell lines (cl4, CB24, and CB36) were noted to have neurite-like structures. Insulin contents of CB cells were high and very similar (Fig. S1B). We also used a transmission electron microscope to observe insulin granules in MIN6-CB4 cells and the original MIN6 cells (Fig. 1D). MIN6-CB4 cells contain significantly more insulin granules than MIN6 cells (Fig. 1D, right graph).

Quantitative RT-PCR analysis showed that these CB cell lines express β-cell-related genes at a level comparable to or higher than MIN6-cl4 cells (Fig. 1E). Interestingly, all of these CB cell lines expressed the *Gcg* and *Sst* genes at variable but lower levels than MIN6-cl4 cells. MIN6-CB4 showed the lowest expression of the *Gcg* and *Sst* genes. The following experiments were performed using the MIN6-CB4 cell line expanded from a frozen stock made at early passage (passage number 4).

### RNA sequencing analysis between MIN6-CB4 and MIN6-cl4 cells

To characterize the newly established MIN6-CB4 cell line, we performed RNA sequencing analysis and compared the results with those of the MIN6-cl4 cell line. Expression levels of the genes related to β-cell function are summarized in Table 1. CB4 cells expressed β-cell-related genes at similar levels to those of MIN6-cl4 cells, but expressed *Ins1*, *Ucn3*, *Pax4*, *Mafb*, *Slc2a2*, and *Ppy* at considerably higher levels than cl4 cells. Higher expression of *Ins1* and *Slc2a2* may suggest that CB4 cells are more β-like cells than cl4 cells. *Ucn3* is known as a β-cell-specific maturation marker^32^. *Pax4* is expressed in a subpopulation of β-cells^33^. *Mafb* is expressed in β-cell precursors, but during β-cell maturation its expression is replaced by the expression of *Mafa*, another large Maf family member^34^. Although *Mafb* expression was higher in CB4 cells than in MIN6-cl4 cells, its expression level was found much lower than that of *Mafa*. In contrast, cl4 cells expressed higher levels of *Gcg* and *Sst*, consistent with the results of quantitative RT-PCR analysis (Fig. 1E). Both the cell lines expressed *Pcsk1*, *Pcsk2*, and *Cpe* encoding processing enzymes, PC1/3, PC2, and CPE, respectively, and *Glp1r* encoding GLP1 receptor at high levels.

**Table 1:** Expression levels of β-cell-related genes in MIN6-CB4 and cl4 cells.

Gene names	CB4	cl4	Ratio
*Ins1*	20,867	6669	3.13
*Ins2*	15,811	16,911	0.93
*Iapp*	19,826	33,660	0.59
*Chga*	5014	3716	1.35
*Pcsk1*	105.0	109.4	0.96
*Pcsk2*	666.4	546.2	1.22
*Cpe*	1015	1620	0.63
*Ucn3*	208.4	70.9	2.94
*Pdx1*	71.2	35.8	1.99
*Pax4*	21.3	0.30	71.0
*Pax6*	42.3	62.0	0.68
*Neurod1*	96.9	49.0	1.98
*Nkx6-1*	53.6	36.3	1.48
*Nkx6-2*	2.18	1.01	2.16
*Isl1*	58.5	66.4	0.88
*Mafa*	80.0	43.1	1.86
*Mafb*	5.22	0.03	174
*Neurog3*	0.22	0	–
*Slc2a1*	26.9	44.3	0.61
*Slc2a2*	261.1	18.9	13.8
*Gck*	20.7	10.4	1.99
*Hk1*	0.25	0.16	1.56
*Abcc8*	171.2	195.5	0.88
*Kcnj11*	51.3	41.9	1.22
*Gcg*	12.0	178.8	0.07
*Ppy*	1.98	0.32	6.19
*Sst*	0	1.48	0
*Glp1r*	72.7	69.9	1.04

The FPKM values from RNA sequencing are shown for the genes listed.

FPKM: fragments per kilobase of exon per million mapped reads.

### Mass spectrometry

**Figure 2:**
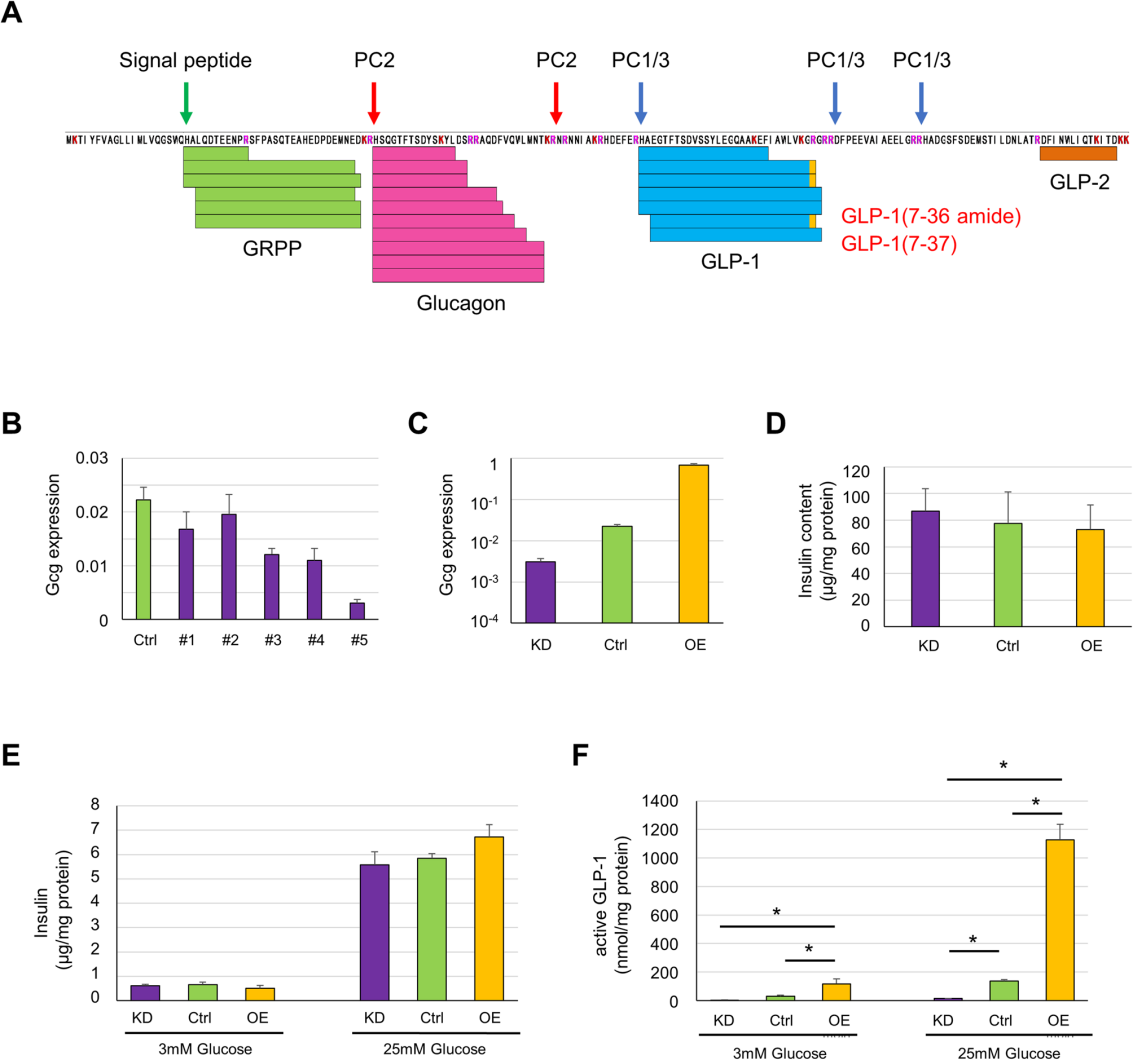
MIN6-CB4 cells with elevated and reduced expression of the *Gcg* gene were produced. (**A**) Mass spectroscopic analysis of preproglucagon proteolytic processing in MIN6-CB4 cells. Peptide-rich fractions (less than 10 kDa) were isolated from the culture supernatant of MIN6-CB4 cells stimulated with 50 mM KCl and were subjected to mass spectroscopy. A total of 24 redundant sequences were mapped to the mouse preproglucagon sequence (GLUC_MOUSE, Uniprot accession entry P55095). Orange boxes indicate C-terminal amidation. Note that GLP-1 (7–36) amide and GLP-1 (7–37) are active forms of GLP-1. (**B**) *Gcg* gene knockdown in MIN6-CB4 cells using lentivirus vectors expressing 5 candidate shRNAs #1–5 for the *Gcg* gene. The expression levels of the *Gcg* gene in knockdown cells were monitored by quantitative RT-PCR. Values are means ± S.D. of technical triplicate. CB4 cells expressing shRNA #5 showed an approximately sevenfold reduction in *Gcg* gene expression. (**C**) Quantitative RT-PCR analysis of *Gcg* gene expression in MIN6-CB4-Gcg^KD^ (KD), CB4-ctrl (Ctrl), and CB4-Gcg^OE^ (OE) cells. Values are means ± S.D. of technical triplicate. (**D**) Insulin content of MIN6-CB4-Gcg^KD^, CB4-ctrl, and CB4-Gcg^OE^ cells. Values are means ± S.D. (n = 6) (**E,F**) Insulin and active GLP-1 secretion from MIN6-CB4-Gcg^KD^, CB4-ctrl, and CB4-Gcg^OE^ cells stimulated with 3 or 25 mM glucose. Active GLP-1 and insulin were secreted in response to glucose concentration. The amount of secreted GLP-1 was almost proportional to the expression levels of the *Gcg* gene in these cells. Values are means ± S.D. (n = 3). *P < 0.05 by Tukey’s test.

Preproglucagon encoded by the *Gcg* gene is first processed to remove the 20-amino acid signal peptide. The resulting proglucagon is expected to be processed to glicentin-related pancreatic polypeptide (GRPP), glucagon, GLP-1 (7–37), GLP-2, etc. in β-cells, considering that they express processing enzymes PC1/3, PC2, and CPE^35^. To confirm the production of these peptides in CB4 cells, we performed mass spectrometric analysis of the peptides smaller than 10 kDa secreted from CB4 cells. As shown in Fig. 2A, we detected GRPP, glucagon, GLP-1 (7–37), and GLP-1 (7–36 amide). However, we could not detect intact GLP-2. It is likely that the amount of GLP-2 produced was lower than our detection limit.

### Overexpression and repression of *Gcg* in MIN6-CB4 cells

MIN6-CB4 cells were considered suitable to examine the effects of altered *Gcg* expression on β-cell function, because these cells expressed only low levels of *Gcg*. In order to alter the levels of *Gcg* expression in MIN6-CB4 cells, we performed overexpression and knockdown of the *Gcg* gene in MIN6-CB4 cells using lentiviral vectors. For *Gcg* knockdown, we examined the effects of 5 candidate shRNAs. As shown in Fig. 2B, expression of Gcg shRNA #5 caused the most effective suppression of Gcg mRNA, and so was used for the subsequent experiments. The resulting *Gcg* overexpressing and knockdown cells were designated CB4-Gcg^OE^ and CB4-Gcg^KD^, respectively. MIN6-CB4 cells infected with an empty lentivirus vector were used as a control (CB4-ctrl). Quantitative RT-PCR analysis showed that the expression levels of *Gcg* in CB4-Gcg^OE^ and CB4-Gcg^KD^ cells were 30.8-fold higher and 7.2-fold lower than those of CB4-ctrl cells, respectively (Fig. 2C). These CB4-derived cells of passage number 10–21 were used for the following experiments.

Insulin content was similar among CB4-Gcg^OE^, CB4-Gcg^KD^, and CB4-ctrl cells (Fig. 2D). We also performed immunofluorescence analysis of these cells using anti-insulin and anti-GLP-1 antibodies. As shown in Fig. S2, these cells were intensely stained with anti-insulin antibody. CB4-Gcg^OE^ cells were clearly stained with anti-GLP-1 antibody, but CB4-ctrl cells were only weakly stained. CB4 cells expressing shRNA #3 were very weakly but positively stained with anti-GLP-1 antibody. CB4-Gcg^KD^ cells expressing Gcg shRNA #5 were almost negative for staining, consistent with the result of quantitative RT-PCR analysis (Fig. 2B,C).

### Active GLP-1 secretion from MIN6-CB4 cells

We examined whether active GLP-1 was secreted from CB4-ctrl, CB4-Gcg^OE^, and CB4-Gcg^KD^ cells in response to glucose. These three cell lines showed similar insulin secretion following the stimulation with 3 mM and 25 mM glucose (Fig. 2E). In the same experiment, we also measured the secretion of active GLP-1. As shown in Fig. 2F, each of these cell lines secreted several-fold more amount of active GLP-1 at 25 mM than at 3 mM glucose. The amount of secreted GLP-1 was roughly proportional to the Gcg gene expression levels among these cell lines (Fig. 2C).

### Effects of *Gcg* expression on insulin secretion of MIN6-CB4 cells

**Figure 3:**
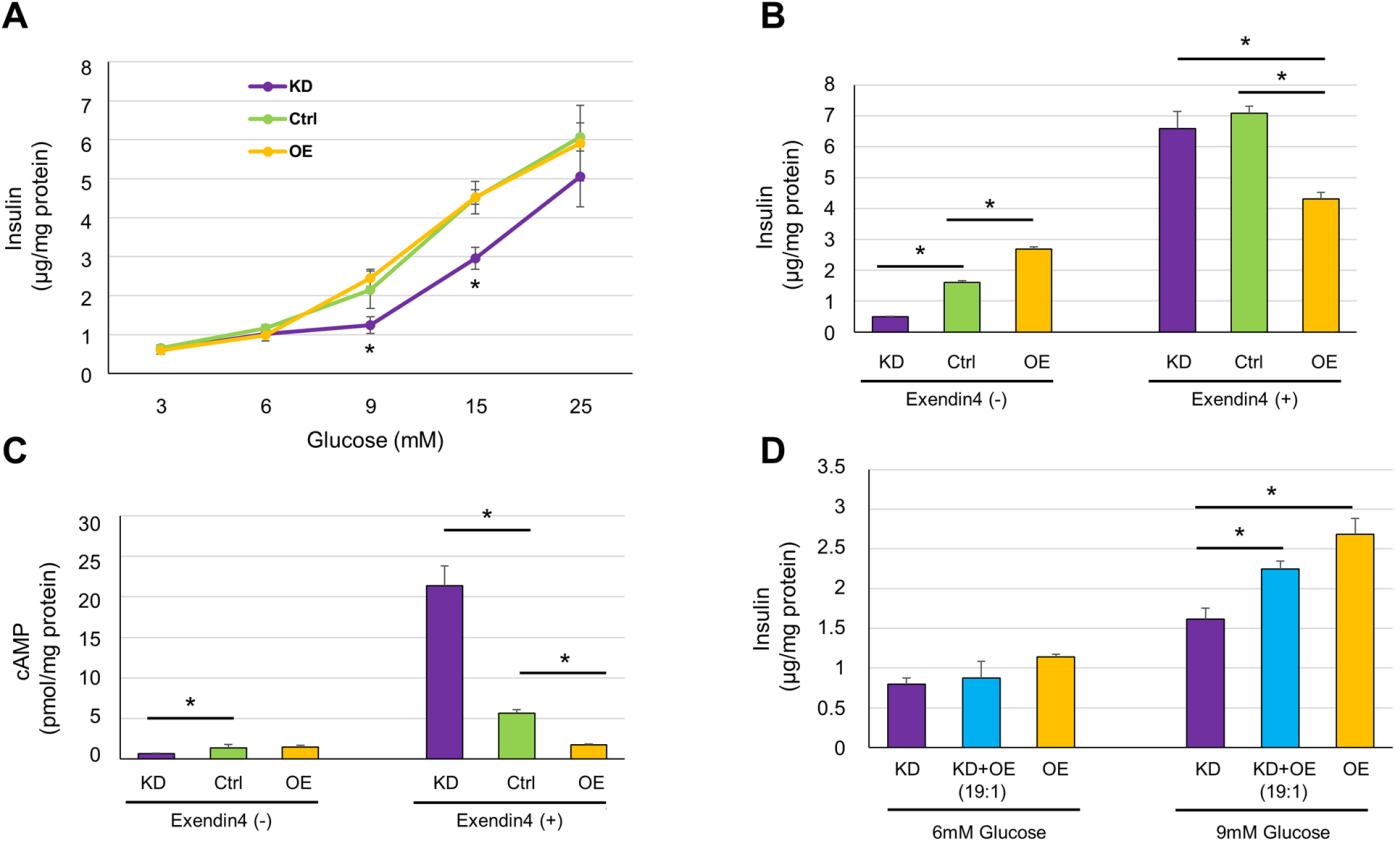
*Gcg* expression levels severely affect the regulation of insulin secretion. (**A**) Insulin secretion from MIN6-CB4-Gcg^KD^, CB4-ctrl, and CB4-Gcg^OE^ cells stimulated with 3, 6, 9, 15, or 25 mM glucose. CB4-Gcg^KD^ cells showed considerably lower secretion than the other cell lines at 9 and 15 mM glucose. Values are means ± S.D. (n = 6). *P < 0.05 by Tukey’s test. (**B**) Potentiation of GSIS by exendin-4. MIN6-CB4-Gcg^KD^, CB4-ctrl, and CB4-Gcg^OE^ cells were stimulated with 9 mM glucose in the presence or absence of 20 nM exendin-4 for 30 min and secreted insulin levels were measured. Values are means ± S.D. (n = 3). *P < 0.05 by Tukey’s test. CB4-Gcg^OE^ cells secreted more insulin than CB4-ctrl cells in the absence of exendin-4 as also seen in Fig. 3A (9 mM glucose). However, the significance level was different (*P* > 0.05 in **A**; *P* < 0.05 in **B**). The reason for this difference is elusive, but it may be due to the difference in the stimulation period (60 min in **A**; 30 min in **B**). (**C**) Cellular cAMP levels after exendin-4 treatment. MIN6-CB4-Gcg^KD^, CB4-ctrl, and CB4-Gcg^OE^ cells were stimulated with 9 mM glucose in the presence or absence of exendin-4 for 30 min and cellular cAMP levels were measured. Values are means ± S.D. (n = 3). *P < 0.05, versus cAMP levels in CB4-ctrl cells by Dunnett’s test. (**D**) Paracrine effect of GLP-1 secreted from CB4-Gcg^OE^ on CB4-Gcg^KD^ cells. CB4-Gcg^OE^ and CB4-Gcg^KD^ cells were mixed at a ratio of 1:19, cultured for 4 days and subjected to GSIS experiment. A small proportion of CB4-Gcg^OE^ cells clearly enhanced the insulin secretion from CB4-Gcg^KD^ cells at 9 mM glucose. Values are means ± S.D. (n = 3). *P < 0.05 by Tukey’s test.

We next examined whether the expression levels of the *Gcg* gene affect GSIS of MIN6-CB4 cells. As shown in Fig. 3A, suppression of *Gcg* gene expression markedly reduced the insulin secretion in response to 9 mM and 15 mM glucose. However, insulin secretion at the other concentrations of glucose (3, 6, and 25 mM) was not significantly affected by *Gcg* expression. Thus, the GSIS dose–response curve was considerably shifted to the right by reducing *Gcg* expression, suggesting that a certain level of basal *Gcg* expression is required for β-cells to maintain proper insulin secretory response to glucose.

### Sensitivity to exendin-4 is reduced by *Gcg* expression

To examine the response of these cell lines to exogenous GLP-1, we tested potentiation of GSIS by exendin-4. In this experiment, we observed the effect of exendin-4 not at 15 mM glucose so far used^36^, but at 9 mM glucose, because the difference in insulin secretion between *Gcg*-expressing CB4-ctrl and CB4-Gcg^OE^ cells and *Gcg*-knockdown CB4-Gcg^KD^ cells was apparent at as low as 9 mM glucose (Fig. 3A). As shown in Fig. 3B, without exendin-4, insulin secretion was increased depending on the expression levels of the *Gcg* gene (see Fig. 3B legend). Exendin-4 enhanced the insulin secretion drastically in CB4-Gcg^KD^ cells and moderately in CB4-ctrl cells. However, its potentiating effect was markedly reduced in CB4-Gcg^OE^ cells. We examined the intracellular cAMP levels of these cells with and without the exendin-4 treatment (Fig. 3C). Without treatment, these cells showed very low cAMP levels and *Gcg* expression appeared to slightly enhance these levels. CB4-Gcg^KD^ cells showed the lowest cAMP level without exendin-4 treatment, but their cAMP level was drastically elevated after treatment. CB4-ctrl cells showed moderate enhancement of the cAMP level after exendin-4 treatment. Contrastingly, CB4-Gcg^OE^ cells exhibited almost negligible elevation of cAMP levels after exendin-4 treatment. Thus, high expression of the *Gcg* gene causes desensitization of GLP-1 receptor, probably due to receptor binding of continuously produced GLP-1, leading to a substantial reduction in cellular response to exogenous agonist.

### Paracrine effects of secreted GLP-1

The above experiments clearly demonstrated that GLP-1 secreted from CB4 cells exerts its effects in an autocrine and/or paracrine manner. To determine whether GLP-1 secreted from CB4 cells causes paracrine effects, CB4-Gcg^OE^ cells were mixed with CB4-Gcg^KD^ cells at a ratio of 1: 19 and cultured for GSIS experiment. We examined insulin secretion at 6 and 9 mM glucose, because the effect of *Gcg* expression on insulin secretion was most clearly observed between these two glucose concentrations (Fig. 3A). As shown in Fig. 3D, addition of 5% of CB4-Gcg^OE^ cells significantly enhanced insulin secretion from CB4-Gcg^KD^ cells under 9 mM glucose stimulation, while insulin secretion with 6 mM glucose stimulation was similar among the three groups (KD, KD + OE, and OE). Thus, the presence of a small proportion of *Gcg*-expressing cells in a β-cell population may greatly improve the insulin secretory response under stimulation with 9 mM glucose.

## Discussion

In the present study, MIN6-CB4, a novel pancreatic β-cell line of the C3B6F1 genetic background, has been established. This cell line was shown to retain differentiated characteristics of β-cells for prolonged culturing periods. RNA sequencing analysis showed that CB4 and MIN6-cl4 cells expressed β-cell-related genes at similar levels. On the other hand, CB4 cells expressed the *Gcg* gene at much lower levels than cl4 cells (Table 1). To examine the effects of different expression levels of the *Gcg* gene on β-cell function, we produced *Gcg*-overexpressing and *Gcg*-knockdown CB4 cells. These cells secreted active GLP-1 at approximately one order of magnitude higher and lower levels than CB4-ctrl cells, respectively (Fig. 2F). Furthermore, in these cells active GLP-1 was demonstrated to be secreted in a glucose-responsive manner (Fig. 2F). GSIS experiments showed that *Gcg*-knockdown severely reduced insulin secretion around a glucose concentration of 9 ~ 15 mM (Fig. 3A). On the other hand, *Gcg*-overexpression did not significantly affect the insulin secretory response to glucose. We also examined the effects of exendin-4 on insulin secretion. Exendin-4 clearly enhanced GSIS in CB4-ctrl and CB4-Gcg^KD^ cells to a similar extent, while exhibiting a weak effect on insulin secretion in CB4-Gcg^OE^ cells (Fig. 3B). The observed difference might be due to the desensitization of GLP-1 receptor by secreted GLP-1. This interpretation was supported by the fact that *Gcg* expression severely suppressed the exendin-4-induced elevation of intracellular cAMP levels (Fig. 3C).

Our study demonstrated that in β-cells a low level of GLP-1 production is critical to maintain a normal insulin secretory response to glucose and exogenous GLP-1. The paracrine effects of GLP-1 produced by β-cells were also demonstrated through GSIS experiment using CB4-Gcg^KD^ cells mixed with a small proportion of CB4-Gcg^OE^ cells (Fig. 3D). Thus, basal signaling through the GLP-1 receptor is essential to maintain a proper insulin secretory response to glucose in CB4 cells. It is curious if this phenomenon is present in islet β-cells in vivo. Consistent with our data, recent studies showed that intraislet GLP-1 and/or glucagon are essential for maintaining proper β-cell responsiveness to glucose and regulating glucose homeostasis^37–41^. Glucagon was shown to bind to GLP-1 receptors and elevate cAMP levels of β-cells. In these studies, intraislet GLP-1 and/or glucagon have been assumed to be derived only from α-cells, but there remains a possibility that part of intraislet GLP-1 and/or glucagon is derived from β-cells.

Accumulating evidence suggests that a subpopulation of islet β-cells express the *Gcg* gene. According to a previous study using nested reverse transcription (RT)-PCR, approximately half of adult β-cells expressed only insulin genes, and the other half expressed multiple islet hormone genes^42^. Approximately 10% of β-cells coexpressed the *Gcg* gene and insulin genes. Recent progress in sequencing technology has enabled single-cell transcriptome analyses. This method has been applied to pancreatic cells^43–46^. In these studies, endocrine cells are clearly classified into four subpopulations (α-, β-, δ-, and PP-cells), but cells identified as β-cells by their gene expression pattern sometimes express the *Gcg* and/or other islet hormone genes at varying levels. Such expression may be explained by residual expression in immature β-cells^47,48^ or may be related to a functional heterogeneity of islet β-cells. So far, it has not been clarified whether or not the coexpression of these endocrine genes affects the function of islet β-cells. While *Gcg* expression in islet β-cells might be low, GLP-1 secreted from a subpopulation of β-cells should immediately bind to the receptors on adjacent β-cells, which might be considered a juxtacrine signaling system. It would be interesting to see if this system is functioning to maintain the blood glucose levels in vivo. This could be investigated by developing and analyzing a β-cell-specific *Gcg* gene knockout mouse model.

It has been previously reported that different MIN6 sublines show different responsiveness to GLP-1^49^. Although the reason for this difference is not known, according to our data, it might be explained by the difference in endogenous *Gcg* expression levels. MIN6-CB4 cells express only low levels of *Gcg* and show long-term stable GSIS and an adequate response to GLP-1. RNA sequencing data also support the notion that MIN6-CB4 cells represent features of mature islet β-cells. Another important property of CB4 cells is long-term stability of β-cell phenotypes, which is essential to establish and analyze genetically manipulated β-cell lines. Thus, MIN6-CB4 cells have distinct advantages over β-cell lines so far reported and will serve as a useful tool for studying the molecular mechanisms of β-cell-specific functions especially related to insulin secretion.

## Materials and methods

### Establishment of stable pancreatic β-cell lines

IT-6 transgenic mice possessing the SV40 T antigen gene under control of the human insulin promoter were maintained on the C57BL/6 background. An IT-6 male mouse was mated with a C3H/HeJ female mouse. The resulting C3B6F1 progeny included a male mouse harboring the IT-6 transgene, which was sacrificed at 9 weeks of age. A number of insulinomas were found in the pancreas of this mouse. Each insulinoma was carefully isolated under a stereomicroscope, mechanically dispersed in a drop of phosphate buffered saline using scissors in a 35-mm dish, and cultured in high-glucose Dulbecco’s modified Eagle’s medium (DMEM) supplemented with 10% heat-inactivated FBS and 0.1 mM β-mercaptoethanol at 37 °C in a humidified atmosphere containing 5% CO_2_. Fifty clones were isolated and designated as MIN6-CB1 to -CB50. Frozen stocks of these clones were made at passage number 4. The original MIN6 cell line^4^ and its subclone MIN6-cl4^14^ were used as controls. All experiments involving animals were carried out in accordance with the institutional guidelines, the ARRIVE (Animal Research: Reporting of In Vivo Experiments) guidelines, and relevant regulations under the protocols #21–089 and #26–066 approved by the Animal Care and Use Committee of Osaka University Graduate School of Medicine.

### Morphological analyses of β-cells

Bright field and fluorescence images of MIN6-CB cells were obtained using a fluorescence microscope (BZ-9000; Keyence, Osaka, Japan). For transmission electron microscopy, the cultured cells were fixed in 2.5% glutaraldehyde at 4 °C for 12 h. The specimens were dehydrated after post-fixation with 1% OsO4 and embedded in Araldite M (Sigma-Aldrich, St. Louis, MO). Semi-thin sections (1 μm) were prepared and treated with 1% toluidine blue. Ultrathin sections were analyzed using the JEOL 1010 transmission electron microscope (JEOL Ltd., Tokyo, Japan).

### Mass spectrometry and data analysis

MIN6-CB4 cells were grown to near confluency in a 10-cm culture dish. The cells were incubated for 30 min at 37 °C in 5 mL Krebs–Ringer bicarbonate buffer (KRBB; 114 mM NaCl, 4.7 mM KCl, 1.2 mM MgSO_4_, 1.2 mM KH_2_PO_4_, 2.5 mM CaCl_2_, 25 mM NaHCO_3_, 10 mM HEPES buffer (pH 7.4), 0.2% BSA) containing 3 mM glucose (low-glucose KRBB). Immediately after washing with low-glucose KRBB, the cells were stimulated with 50 mM KCl in low-glucose KRBB for 10 min. The supernatant was collected in a pre-chilled tube before it was subjected to solid phase extraction, and separated by gel filtration to obtain peptide-rich fractions (less than 10 kDa) as previously described^50^. Samples were lyophilized and stored for mass spectrometric analysis. Lyophilized samples were reconstituted in 2% acetonitrile and 0.1% formic acid. Constituent peptides were identified by liquid chromatography tandem mass spectrometry, which was conducted on a NanoFrontier system (Hitachi, Tokyo, Japan) connected to an Orbitrap XL mass spectrometer (Thermo Fisher, Waltham, MA)^50^. Approximately one twentieth of the sample solutions each corresponding to a 10-cm dish was analyzed in triplicate. Acquired tandem mass spectra were searched against the SwissProt mouse database using Mascot software version 2.5.1 (Matrix Science, London, U.K.; https://www.matrixscience.com) with a precursor mass tolerance of 2 ppm and a product ion mass tolerance of 0.05 Da, with no enzyme specified. C-terminal amidation, N-terminal pyroglutamylation, and methionine oxidation were considered as possible post-translational modifications. A sequence was considered as identified if it meets the Mascot identity threshold (*P* = 0.05) as described^50^.

### Measurement of insulin and GLP-1 secretion and insulin content

MIN6-CB cells were cultured in 24-well plates (5 × 10^5^ cells/well) for 4 days. Cells were starved in low-glucose KRBB for 30 min. After washing twice with the same buffer, the cells were then incubated in KRBB with 3, 6, 9, 15, or 25 mM glucose or with 15 mM glucose in the presence of 30 mM KCl, 10 nM somatostatin, or 20 nM exendin-4 for 1 h. The supernatant was collected from each well and insulin secretion was measured using an ELISA kit (Mercodia, Uppsala, Sweden); values were normalized to the protein content of each well. Similarly, secreted active GLP-1 was measured using an ELISA kit (Immuno-Biological Lab, Gunma, Japan). To measure insulin content, cells cultured in a 6-cm dish were collected. Half of the cells were subjected to acid–ethanol extraction and assayed for insulin using an ELISA kit. The other half of the cells were lysed in RIPA buffer and measured for protein concentration using protein assay dye reagent (Bio-Rad, Hercules, CA).

### Lentiviral vector construction and infection

To produce a lentiviral vector expressing the *Gcg* gene, the CMV-GFP cassette of pCS-CDF-CG-PRE was replaced with the PGK promoter-driven *Gcg* cDNA. The IRES-puromycin-resistance gene cassette was inserted down-stream of the *Gcg* cDNA sequence. To produce the lentivirus vector, HEK293T cells were transfected with the above plasmid together with two packaging plasmids (pCMV-VSV-G-RSV-Rev and pCAG-HIVgp)^51,52^. Empty lentivirus vector was also produced and used as a control. MIN6-CB4 cells were dissociated with trypsin–EDTA, suspended in culture medium, and infected with the Lenti-Pgk-Gcg-IRES-puro vector or an empty lentivirus vector at an approximate multiplicity of infection of 8. The cells were then cultured and selected with 1.2 μg/mL of puromycin for 2 weeks. The resulting colonies were pooled and cultured for use in the experiments.

### Knockdown of the *Gcg* gene

Five shRNA sequences targeting the *Gcg* gene were obtained from the MISSION LentiPlex Mouse Pooled shRNA Library (Sigma-Aldrich) for evaluation. The complementary oligonucleotides corresponding to these sequences were hybridized and placed under the mouse U6 (mU6) promoter in the lentiviral vector plasmid for shRNA expression^36^. In this plasmid, the PGK promoter-driven puromycin-resistance gene was inserted upstream of the mU6 promoter in the opposite direction and used as a selection marker. The shRNA-expressing lentiviral vectors were generated in HEK293T cells transfected with the above plasmids together with packaging plasmids as described above. MIN6-CB4 cells were infected with the resulting lentivirus vectors at an approximate multiplicity of infection of 10 and cells were selected with 2.0 μg/mL puromycin for 2–3 weeks. The resulting colonies were pooled and cultured for use in the experiments. The shRNA oligonucleotide pair that were capable of effective knockdown were: shGcg-5F: GATCCCCGGGCATTGTGTAACCCAACGATTCTCGAGAATCGTTGGGTTACACAATGCTTTTTGT and shGcg-5R: CTAGACAAAAAGCATTGTGTAACCCAACGATTCTCGAGAATCGTTGGGTTACACAATGCCCGGG.

### Quantitative PCR analysis

Total RNA was extracted from MIN6-CB cells by the acid guanidinium-phenol-chloroform (AGPC) method or using an RNA purification kit (NucleoSpin RNA Plus; Macherey-Nagel, Düren, Germany). cDNA synthesis was performed using ReverTra Ace-α (Toyobo, Tokyo, Japan). The cDNA was subjected to quantitative PCR on an ABI 7300 or StepOnePlus real-time PCR system, using SYBR Premix Ex Taq (Takara, Shiga, Japan) or FastStart SYBR Green Master (Roche Diagnostics, Indianapolis, IN). PCR was performed with an initial step of 10 s for SYBR Premix Ex Taq or 10 min for FastStart SYBR Green Master at 95 °C followed by 40 cycles of 5 s at 95 °C and 31 s at 60 °C. Standard curves were generated separately for each target gene and the β-actin (*Actb*) gene. The expression level of each gene was normalized to that of *Actb*. The primers used in this study are listed in Table S1.

### RNA sequencing and differential expression analysis

Total RNA was extracted from MIN6-CB4 and MIN6-cl4 cells using an RNA purification kit (NucleoSpin RNA Plus). The quality of the purified total RNA was examined using an Agilent 2100 Bioanalyzer (Agilent, Santa Clara, CA). PolyA^+^ RNA was isolated and fragmented. Sequencing libraries were prepared from the fragmented RNA using the TruSeq Stranded mRNA Sample Prep Kit (Illumina, San Diego, CA). The paired-end libraries had a peak fragment size of approximately 450 bp and were analyzed on a HiSeq 2500 genome analyzer (Illumina) with a 100-base paired-end read setting. Approximately 50 million reads per sample were mapped against the mouse mm10 (GRCm38) reference assembly. The expression levels of individual transcripts and genes were estimated with Cufflinks.

### Measurement of insulin secretion and cAMP content

MIN6-CB4 cells were cultured in 6-well plates (2 × 10^6^ cells/well) for 4 days. Cells were starved in low-glucose KRBB for 30 min. After washing twice with the same buffer, the cells were then incubated in KRBB containing 9 mM glucose in the presence or absence of 20 nM exendin-4 for 30 min. The supernatant was collected from each well and insulin secretion was measured. Cellular cAMP levels were determined using a commercial kit (Cyclic AMP Select ELISA Kit; Cayman, Ann Arbor, MI) according to the manufacturer’s instructions.

### Statistical analysis

Statistical analyses were carried out by Student’s t-test or by Dunnett’s test or Tukey’s test using the open-source statistical analysis software MEPHAS version 1.1 (https://alain003.phs.osaka-u.ac.jp/mephas/index.html)^53^. A value of *P* < 0.05 was considered statistically significant.

## Supplementary Information

Supplementary Information.
