# Supplementary material for: Establishment of a long-term stable β-cell line and its application to analyze the effect of Gcg expression on insulin secretion
Source: Sci Rep. 2021 Jan 12;11:477. doi: 10.1038/s41598-020-79992-7 (PMC7804151; doi:10.1038/s41598-020-79992-7)

Supplementary Material

Establishment of a long-term stable β-cell line and its application to analyze the effect of *Gcg* expression on insulin secretion

Satsuki Miyazaki[1], Fumi Tashiro[1], Takashi Tsuchiya[2], Kazuki Sasaki[2] & Jun-ichi Miyazaki[3]

[1]Division of Stem Cell Regulation Research, Center for Medical Research and Education, Osaka University Graduate School of Medicine, Suita, Osaka, Japan [2]National Cerebral and Cardiovascular Center, Suita, Osaka, Japan [3]The Institute of Scientific and Industrial Research, Osaka University, Ibaraki, Osaka, Japan

Table S1. Primers used for quantitative PCR.

Gene	Forward primer (5' to 3')	Reverse primer (5' to 3')	Reference
Ins1	GCCCTTAGTGACCAGCTATAATCAG	GGGTAGGAAGTGCACCAACAG	Lee, E.M. *et al*.
Ins2	GCTTCTTCTACACACCCATGTC	AGCACTGATCTACAATGCCAC	PrimerBank ID 6680463a1
Slc2a2	AAGGATCTGCTCACATAGTCACT	TTGCAGCCAACATTGCTTTGA	Du, D. *et al*.
Hk1	GACCCGAGGCATCTTCGA	AGCAGCGCTAATCGGTCACT	Miyazaki, S. *et al*.
Gck	TGAGCCGGATGCAGAAGGA	GCAACATCTTTACACTGGCCT	PrimerBank ID 31982798a1
Kcnj11	AAGGGCATTATCCCTGAGGAA	TTGCCTTTCTTGGACACGAAG	PrimerBank ID 6754426a1
Slc2a1	CAGTTCGGCTATAACACTGGTG	GCCCCCGACAGAGAAGATG	PrimerBank ID 22094111a1
Gcg	TTACTTTGTGGCTGGATTGCTT	AGTGGCGTTTGTCTTCATTCA	PrimerBank ID 33468853a1
Sst	ACCGGGAAACAGGAACTGG	TTGCTGGGTTCGAGTTGGC	PrimerBank ID 6678035a1
Actb	AGTGTGACGTTGACATCCGTA	GCCAGAGCAGTAATCTCCTTCT	PrimerBank ID 6671509a3

PrimerBank: https://pga.mgh.harvard.edu/primerbank/

Lee, E.M. *et al*. *Islets* **10**, 25-39 (2018).

Du, D. *et al*. *Mol. Biol. Rep.* **37**, 1867-1874 (2010).

Miyazaki, S. *et al*. *Diabetes* **59**, 2854-2861 (2010).

Figure S1. Glucose-stimulated insulin secretion from MIN6-CB cell clones. (**A**) Fifty insulinomas were isolated from an IT6 transgenic mouse on the C3B6F1 background and were independently cultured for approximately 2 months. Twelve clones were removed because of fibroblast contamination or poor growth. Insulin secretion from each clone stimulated with 3 or 25 mM glucose was measured. Values are means (n = 2). (**B**) Insulin content of MIN6-CB cell lines (#3, #4, #23, #24, and #36). Values are means (n = 2).

A

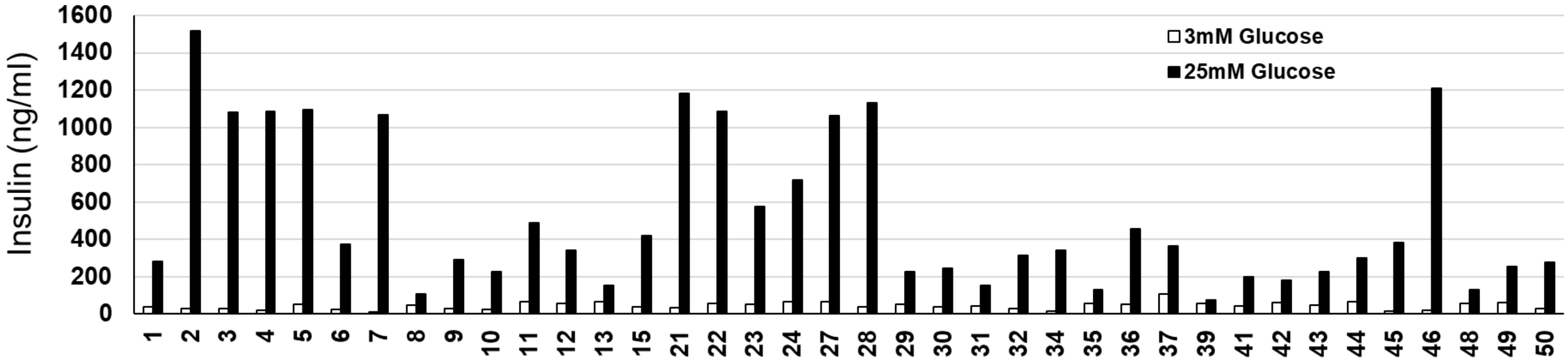

B

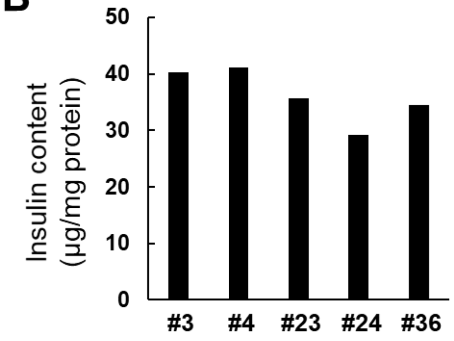

Figure S2. Immunocytochemical analysis of insulin and GLP-1 expression in MIN6-CB4-Gcg^{KD} cells expressing Gcg shRNA #3 or #5, CB4-ctrl, and CB4-Gcg^{OE} cells. The cells were washed with PBS, fixed in 4% paraformaldehyde for 10 min, washed again with PBS, and incubated in PBS with 1% Triton X-100 for 5 min. After washing with PBS, the cells were treated with blocking reagent (Blocking One; Nacalai Tesque, Kyoto, Japan). For immunostaining, the samples were incubated with mouse anti-GLP-1 monoclonal antibody (HYB147-12; Santa Cruz, Dallas, TX) or guinea pig anti-insulin antibody (Dako, Carpinteria, CA) for 1 h at room temperature, washed with PBS, and then incubated with Alexa Fluor 488-conjugated goat anti-mouse IgG1 or Alexa 647-conjugated goat anti-guinea pig IgG (Molecular Probes, Eugene, OR) for 1 h at room temperature. The slides were counterstained with DAPI. Scale bars, 100 μm.

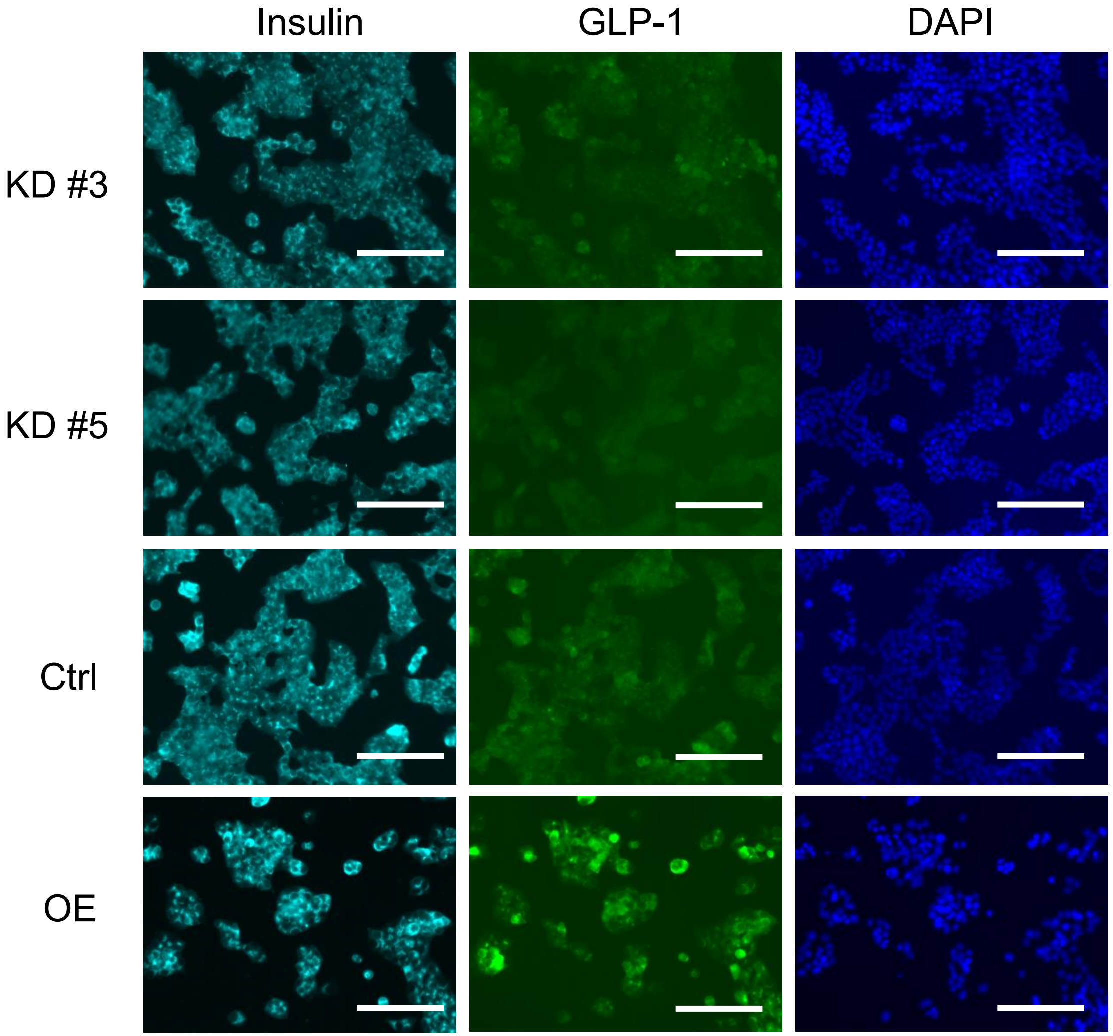

Supplement: Supplementary file 1 — Supplementary Information. [file 41598_2020_79992_MOESM1_ESM.pdf]
